# Near-Infrared Spectroscopy – Electroencephalography-Based Brain-State-Dependent Electrotherapy: A Computational Approach Based on Excitation–Inhibition Balance Hypothesis

**DOI:** 10.3389/fneur.2016.00123

**Published:** 2016-08-08

**Authors:** Snigdha Dagar, Shubhajit Roy Chowdhury, Raju Surampudi Bapi, Anirban Dutta, Dipanjan Roy

**Affiliations:** ^1^Cognitive Science Lab, International Institute of Information Technology, Hyderabad, India; ^2^School of Computing and Electrical Engineering, Indian Institute of Technology, Mandi, India; ^3^School of Computer and Information Sciences, University of Hyderabad, Hyderabad, India; ^4^Leibniz-Institut für Arbeitsforschung an der TU Dortmund, Dortmund, Germany; ^5^Centre of Behavioral and Cognitive Sciences, University of Allahabad, Allahabad, India

**Keywords:** tDCS, balance of exc and inh, neural mass model, somatosensory cortex, neural plasticity

## Abstract

Stroke is the leading cause of severe chronic disability and the second cause of death worldwide with 15 million new cases and 50 million stroke survivors. The poststroke chronic disability may be ameliorated with early neuro rehabilitation where non-invasive brain stimulation (NIBS) techniques can be used as an adjuvant treatment to hasten the effects. However, the heterogeneity in the lesioned brain will require individualized NIBS intervention where innovative neuroimaging technologies of portable electroencephalography (EEG) and functional-near-infrared spectroscopy (fNIRS) can be leveraged for Brain State Dependent Electrotherapy (BSDE). In this hypothesis and theory article, we propose a computational approach based on excitation–inhibition (E–I) balance hypothesis to objectively quantify the poststroke individual brain state using online fNIRS–EEG joint imaging. One of the key events that occurs following Stroke is the imbalance in local E–I (that is the ratio of Glutamate/GABA), which may be targeted with NIBS using a computational pipeline that includes individual “forward models” to predict current flow patterns through the lesioned brain or brain target region. The current flow will polarize the neurons, which can be captured with E–I-based brain models. Furthermore, E–I balance hypothesis can be used to find the consequences of cellular polarization on neuronal information processing, which can then be implicated in changes in function. We first review the evidence that shows how this local imbalance between E–I leading to functional dysfunction can be restored in targeted sites with NIBS (motor cortex and somatosensory cortex) resulting in large-scale plastic reorganization over the cortex, and probably facilitating recovery of functions. Second, we show evidence how BSDE based on E–I balance hypothesis may target a specific brain site or network as an adjuvant treatment. Hence, computational neural mass model-based integration of neurostimulation with online neuroimaging systems may provide less ambiguous, robust optimization of NIBS, and its application in neurological conditions and disorders across individual patients.

## Introduction

Stroke or cerebrovascular accident is caused when an artery carrying blood from heart to an area in the brain bursts or a clot obstructs the blood flow thereby preventing delivery of oxygen and nutrients. It is the most debilitating consequence of cardiovascular disorder with over 50 million stroke survivors worldwide. It is also the second leading cause of dementia after Alzheimer’s disease. The projected cost of patient care for stroke will reach trillions of dollars over the next five decades. Therefore, innovative methodologies for restorative neurorehabilitation are urgently required to reduce long-term disability. Here, the ability of the central nervous system to respond to intrinsic or extrinsic stimuli by reorganizing its structure, function, and connections can be leveraged, which is called *neuroplasticity*. Neuroplasticity is involved in poststroke restorative rehabilitation but also can cause maladaptive functional outcomes, which can compromise regain of function *via* implementation of sub-optimal compensatory strategies. Beneficial neuroplasticity may be facilitated with carefully designed non-invasive brain stimulation (NIBS) protocols, which have been shown to modulate brain and spinal (1) network interactions. Indeed, appropriately focused NIBS protocol may facilitate learning and consolidation during rehabilitative training (2) where transcranial direct current stimulation (tDCS), a NIBS modality, uses low direct currents to modulate cortical excitability and has been found to be a promising tool to facilitate neuroplasticity during stroke rehabilitation (3). During stroke rehabilitation, it is also important to enforce normative movement coordination and sensory feedback and penalize maladaptive compensatory movements (4), which can be achieved with neuromuscular electrical stimulation (NMES). Here, the hypothesis on volitionally (electromyogram, EMG) driven NMES for stroke rehabilitation is based on sensorimotor integration theory, which states that sensory input from movement of the affected limb directly influences subsequent motor output from the brain where alternative motor pathways can be recruited and activated to assist the stroke-damaged motor output pathways (5). Indeed, it was found that EMG-driven NMES effected greater brain cortical perfusion than voluntary muscle contraction or NMES alone (6) where the beneficial brain activation can be further facilitated with tDCS (7) toward beneficial neuroplasticity. Therefore, it is postulated that a tDCS in conjunction with peripheral electrical stimulation will modulate the activity in the dysfunctional network, to restore an adaptive equilibrium in a disrupted network for optimal behavioral outcome, and suppress maladaptive plastic changes for functional advantage *via* their synergistic effect on task–relevant neuronal activation patterns. Recent studies also suggest that the endogenous state of cortical activity that is dependent on individual physiology of the brain as well as psycho-physiological factors can alter the effects and efficacy of poststroke tDCS treatment (8). These studies showed that the evidence for therapeutic efficacy is still uncertain since the treatment effects of tDCS in patients with stroke are rather inconsistent across studies. This is expected poststroke, specifically in acute and sub-acute stages, when the heterogeneous acute regional imbalance of brain’s E–I balance, for example due to glutamate surge, may be going through a dynamic reorganization based on the available “structural reserve,” i.e., the integrity of the white matter pathways. The consequent mal-adaptive neuroplastic alterations of cortical activation and excitability and their global impact in sub-acute and chronic stages are therefore subject-specific, which determine the individual endogenous state of cortical activity. Although, tDCS is already used in stroke rehabilitation, however, currently available “one-size-fits-all” methods for planning tDCS intervention limits its clinical translation due to inter-subject variability and lack of intra-subject reliability. Here, understanding of individual interactions between the endogenous brain states and therapeutic mechanisms can lend to tDCS interventions to antagonize subject-specific maladaptive alterations by the regulation of cortical excitability, which may then lead to beneficial plasticity that is crucial for re-installing efficient information transfer in the brain during neurorehabilitation. This may be feasible with a computational model based on neuroimaging that can objectively monitor individual brain state during NIBS that can then be used to adjust stimulation protocols accordingly (9).

In this hypothesis and theory article, we present an E–I based brain model to objectively quantify the individual brain state poststroke, which can then guide the planning of tDCS as an adjuvant treatment (to physical therapy and/or pharmacotherapy). The dysregulation of cortical excitability during acute and sub-acute stroke is a characteristic feature of unbalanced network, which may lead to symptoms depending on the area(s) in which the imbalance occurs. Here, we present innovative technologies of portable functional near-infrared spectroscopy (fNIRS) and electroencephalography (EEG) neuroimaging systems to objectively build the E–I based poststroke brain model that can be used to guide and quantify the progress of tDCS treatment regime. Here, E–I balance hypothesis implements homeostatic regulation of cortical E–I to allow efficient information transfer using multi-focal tDCS. It is postulated that tDCS leads to a rapid dynamic variations of the brain cell microenvironment (10) that perturbs hemodynamic (fNIRS) and electrophysiological (EEG) responses. The interactions between the hemodynamic and electrophysiological responses, captured with NIRS–EEG joint imaging, may provide an assessment of underlying E–I balance. Also, while the plastic changes occur during neurorehabilitation, the adjuvant tDCS treatment is planned such that the network maintains a certain amount of stability in order to produce meaningful output (11). In Section “Evidence in Support of Alterations in Cortical Excitation–Inhibition with Non-invasive Cortical and Peripheral Electrical Stimulation,” we give a summary of the evidence in support of post-tDCS perturbation of E–I balance from EEG, fMRI, and computational studies. In Section “Computational E–I Modeling using fNIRS–EEG Joint-Imaging During tDCS,” we propose biophysical “forward models” of the alterations in neural and hemodynamic responses due to tDCS perturbation. In Section “E–I Balance Hypothesis for Brain-State Dependent Electrotherapy in Stroke Rehabilitation,” we propose a new framework for online brain-state dependent electrotherapy during poststroke neurorehabilitation along with pilot data and simulations. Section “Discussion” summarizes and concludes with future directions.

## Evidence in Support of Alterations in Cortical Excitation–Inhibition with Non-Invasive Cortical and Peripheral Electrical Stimulation

### Evidence from EEG–fMRI Studies with NIBS

A common practice that is followed during NIBS is the application is restricted to an isolated brain area. Although isolated brain areas are targeted during non-invasive stimulation, it affects multiple local as well as distant brain areas of the cerebral cortex in essence impacting cognitive networks (12, 13). We suggest that the regional cortical E–I balance, measured by ratios of glutamate/GABA, and local oscillations (representing intrinsic brain state) (14) are crucial for meaningful interpretations of individual cognitive performance and deficits than glutamate and/or GABA alone. This formulation finds support from evidences where E/I balance plays a major role in normal cognition, as well as the symptomatic patterns of a variety of clinical conditions (11). Although abnormality in E/I balance is perhaps critical for mechanistically understanding neurocognitive disorders, there is a genuine gap in understanding the role of E/I balance in conjunction with variety of well accepted (tDCS) or not so well accepted transcranial brain stimulation techniques (15). We posit that by combining computational methods, NIBS, and neuroimaging, in principle, we can bridge that gap and provide a detailed mathematical framework for understanding the impact of E/I balance for brain networks where reorganization takes place following plasticity.

### Evidence from EEG–fNIRS Studies with NIBS

**Figure 1:**
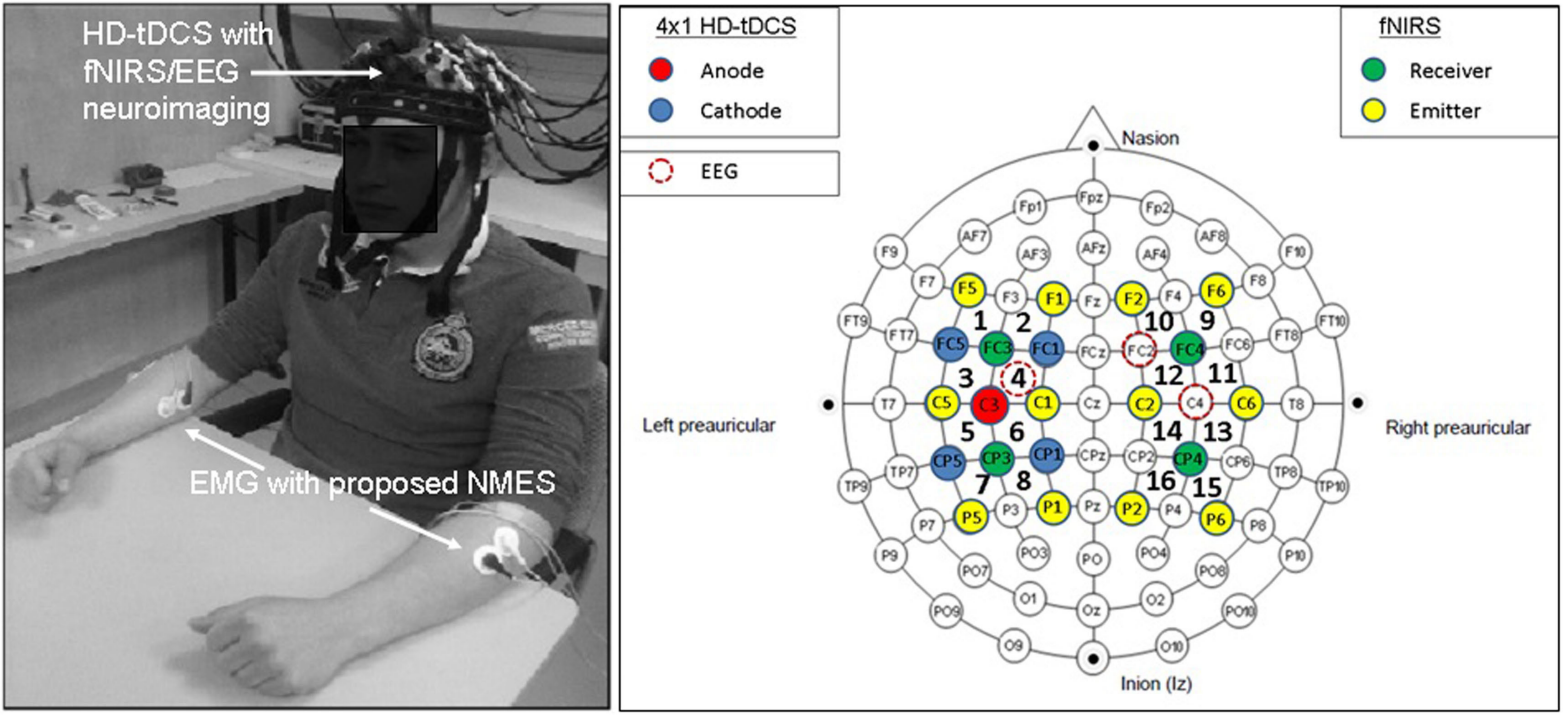
**Electroencephalography (EEG)-functional near-infrared spectroscopy (fNIRS) joint-imaging during non-invasive brain stimulation (NIBS) and neuromuscular electrical stimulation (NMES)**. Left panel: experimental setup for the upper limb, Right panel: EEG–fNIRS sensor montage along with NIBS stimulation electrodes [from Ref. (20)]. Figure adapted from Dutta et al. (19).

**Figure 2:**
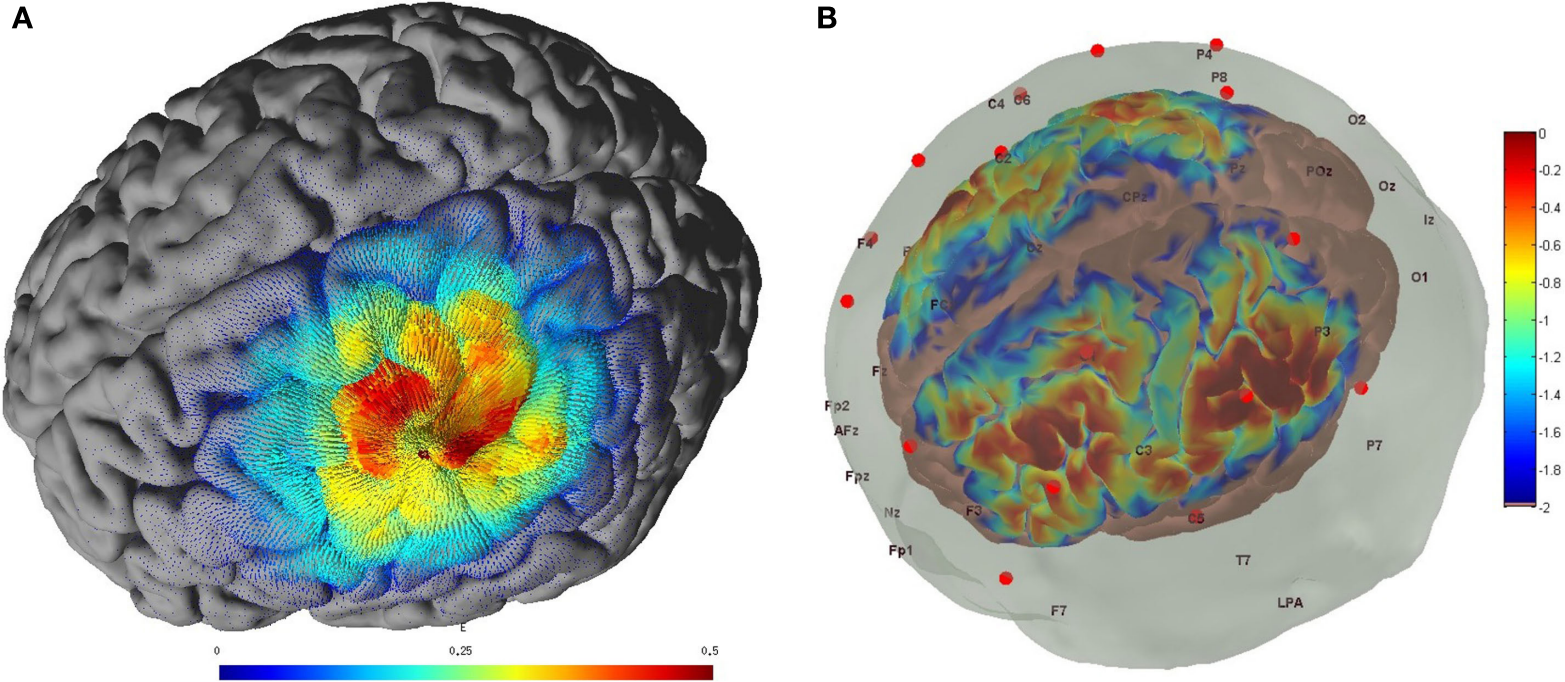
**(A)** Electric field estimated at the gray matter surface due to 2-mA HD-tDCS (electrode montage shown in Figure 1). **(B)** Measurement sensitivity distribution of the fNIRS probe (montage shown in Figure 1) at gray matter surface.

Non-invasive brain stimulation, e.g., tDCS (3), can lead to alterations in both the cortical neural activity and the hemodynamics (16) that are related by neurovascular coupling (NVC). The EEG can record the potential at the scalp due to the electric currents from all excitable membranes of the brain tissue (17). To measure the hemodynamics, near-infrared spectroscopy (NIRS) sensor contains light source in the near-infrared (NIR) wavelength range (700–1300 nm) that biological tissue is relatively transparent to and can penetrate into the superficial brain (1–3 cm penetration depth) (18). In the blood supply to the brain tissue, hemoglobin’s constituents – oxyhemoglobin and deoxyhemoglobin – exhibit distinct absorption spectra (i.e., distinct chromophore) in the NIR range. Here, the brain tissue serves as the scattering medium that allows NIR light detectors placed on the scalp to help estimate the chromophore’s (in both arterial and venous blood) absorption using the Beer- Lambert law (18). Therefore, while EEG provides an electrophysiological measure of cortical neural activity, functional NIRS provides a measure of the related hemodynamic component that supplies glucose *via* NVC. Figure 1, adapted from Dutta et al. (19), shows an illustrative experimental setup. An eight-channel dual tDCS–EEG system (StarStim, Neuroelectrics, Spain) was used to deliver constant direct current (2 mA for 20 min) to the left sensorimotor cortex (SMC) *via* a 4 × 1 anodal High-Definition-tDCS (HD-tDCS) electrode montage with the anode at C3 (red circle in Figure 1) and surrounding four cathodes as return electrodes (blue circles in Figure 1). EEG was recorded at 500 Hz from all the eight electrodes before “Pre” and after “Offline,” and only from three non-stimulation electrodes during “Online” (C1, C4, FC2). A multi-channel fNIRS system (Oxymon MK III, Artinis Medical Systems, Netherlands) was used to continually measure the cortical hemodynamic changes from 16 channels at 10 Hz, represented by receiver (green circles in Figure 1) – emitter (yellow circles in Figure 1) combination covering the left (Channels 1–8) and right (Channels 9–16) hemisphere. EMG was measured from the left and right hand finger flexors and extensors at 2000 Hz (PowerLab, ADInstruments, USA). Prior work has shown that HD-tDCS reduced bilateral activation of primary SMC during task performance during and after tDCS (20). Here, it is important to quantify the interactions between motor training and NIBS in order to determine their relative timing as well as to optimize the NIBS protocol as an adjuvant treatment in poststroke rehabilitation. We have shown with a computational pipeline (16) that tDCS-evoked cortical neural activity and hemodynamics can be monitored with NIRS–EEG joint imaging. The electric field in the gray matter surface during 4 × 1 anodal HD-tDCS with 2 mA constant direct current is shown in Figure 2A while the measurement sensitivity distribution of fNIRS probe at gray matter surface is shown in Figure 2B. Here, the EEG measurement sensitivity analysis using the Laplacian spatial filter consisting of HD-tDCS electrodes in “Offline” condition is related to the HD-tDCS electric field/current density modeling, as discussed in Guhathakurta and Dutta (16). We postulate that tDCS may non-specifically alter the synaptic activity of the excitatory pyramidal neurons (ePN) at a population level (21) where glutamate activates patterns of calcium signaling for opposing control of inhibitory GABA synapses (22) thereby regulating the E-I balance by the balance of excitatory and inhibitory synaptic currents (23) which have been found to promote efficient coding and metabolic efficiency (24). Here, EEG–NIRS-based joint imaging can be used to assess NVC during the application of anodal tDCS (25) using intrinsic mode functions (IMFs) for fNIRS and EEG time-series (16). However, the challenge remains in capturing mostly non-linear spatiotemporal interactions between the cortical neural activity and the hemodynamics (26), which may be possible with an E-I brain model (27) based on EEG–NIRS joint-imaging during tDCS.

### Evidence from fMRI and fNIRS Studies with Peripheral Electrical Stimulation

Neurophysiological research has shown that repetitive electrical stimulation of the common peroneal nerve elicits lasting changes in corticospinal excitability, possibly as a result of co-activating motor and sensory fibers (28). In fact, primary sensory and motor cortex excitability have been found to be co-modulated in response to peripheral electrical stimulation (29). This may be due to cortico-cortical projections between primary sensory and motor cortex, and this mechanism may underpin changes in corticomotor excitability in response to afferent input generated by peripheral electrical nerve stimulation. Moreover, Khaslavskaia and Sinkjaer (30) showed in humans that concurrent motor cortical drive present at the time of stimulation enhanced motor cortical excitability. This leads to the exciting possibility that peripheral electrical stimulation could be used to drive cortical plasticity during stroke rehabilitation (31), possibly in conjunction with NIBS (32). Here, the cerebral activation patterns during peripheral electrical stimulation can be elucidated with fMRI (33) and fNIRS (34). The fMRI study (33) revealed peripheral electrical stimulation-related activation pattern comprising the contralateral primary motor cortex, primary somatosensory cortex and premotor cortex; the ipsilateral cerebellum; bilateral secondary somatosensory cortex, the supplementary motor area, and anterior cingulate cortex. Also, a greater bilateral sensorimotor network activation profile with high current intensities was attributed to an increased bilateral sensorimotor integration (34). However, dosing of peripheral electrical stimulation remains a challenge because of non-linear effects where stimulation above motor threshold increased cortical excitability while stimulation below motor threshold, but sufficient to induce sensory perception, produced conflicting results (31). Moreover, there appeared to be time effects, where longer periods of stimulation induced more sustained changes in cortical excitability. Therefore, a computational framework based on neuroimaging is necessary to delineate these effects and computational modeling approaches are explored in the next section.

## Computational E–I Modeling Using fNIRS–EEG Joint-Imaging during tDCS

One of the major goals of electrical stimulation techniques is to provide capacity of neurorehabilitation by facilitating movement (with peripheral electrical stimulation) and modulating the brain plasticity in specific brain areas (with NIBS). It is generally agreed upon that cortical stimulation alters excitability in specific cortical areas so as to enhance plasticity in various motor, perceptual tasks. Many approaches often do not consider that brain is a dynamical system with the amount of plasticity and functional connectivity well regulated in specific brain areas in an activity-dependent manner. These activity levels, being persistent, do not show signs of adaptation and the connectivity patterns dynamically change mostly in an unpredictable manner. In order to look at the persistent activity levels, one can focus on underlying synaptic changes as has been recently carried out in a computational study by Sigala et al. (14). We preview here what could be considered as a dynamic consequence of E–I parameter variation and ongoing modulation of power, amplitude and firing rate of multiunit activity (MUA), or neural population activity as measured by EEG. Oscillations are important markers of neural state activity during sensori-motor plasticity and behavior (35, 36). Increasing excitatory–excitatory (EE) synaptic connectivity has the potential to increase firing rate without any bound. Hence, it is necessary to simultaneously adjust excitatory–inhibitory (EI) synaptic strength or inhibitory–excitatory (IE) in order to compensate for excess excitation received by cortical population in the targeted brain area. In general, as has been understood, NIBS would result in an increase in the firing rate (in Hertz) in the targeted brain area. This excess may destabilize the network and results in abnormal oscillations indicated by the neural state. There, the appropriate feedback inhibition must be applied in order to bring endogenous oscillations under control and to produce stable cortical output from the targeted brain regions (27). This is certainly possible to achieve given the thalamocortical neural mass model (NMM) proposed in this work where observed lasting changes are attributed to the lasting modulation of the E–I balance. We have also enriched the thalamocortical NMM to investigate cortico-muscular coherence during anodal tDCS in conjunction with volitional muscle activation and/or peripheral electrical stimulation, by adding a resonant spinal-musculoskeletal system driven by cortical NMM that fed back to the thalamic NMM *via* sensory afferents (21). This was based on the hypothesis that perturbations from ePN to the spinal-musculoskeletal system can be shaped by its resonant properties (21), which can feed back to the relay nucleus of the thalamus *via* sensory afferents and create coherence between thalamus, cortex, and muscle in human.

### Neural Mass Model for Capturing Neuronal Response to tDCS Using EEG

**Figure 3:**
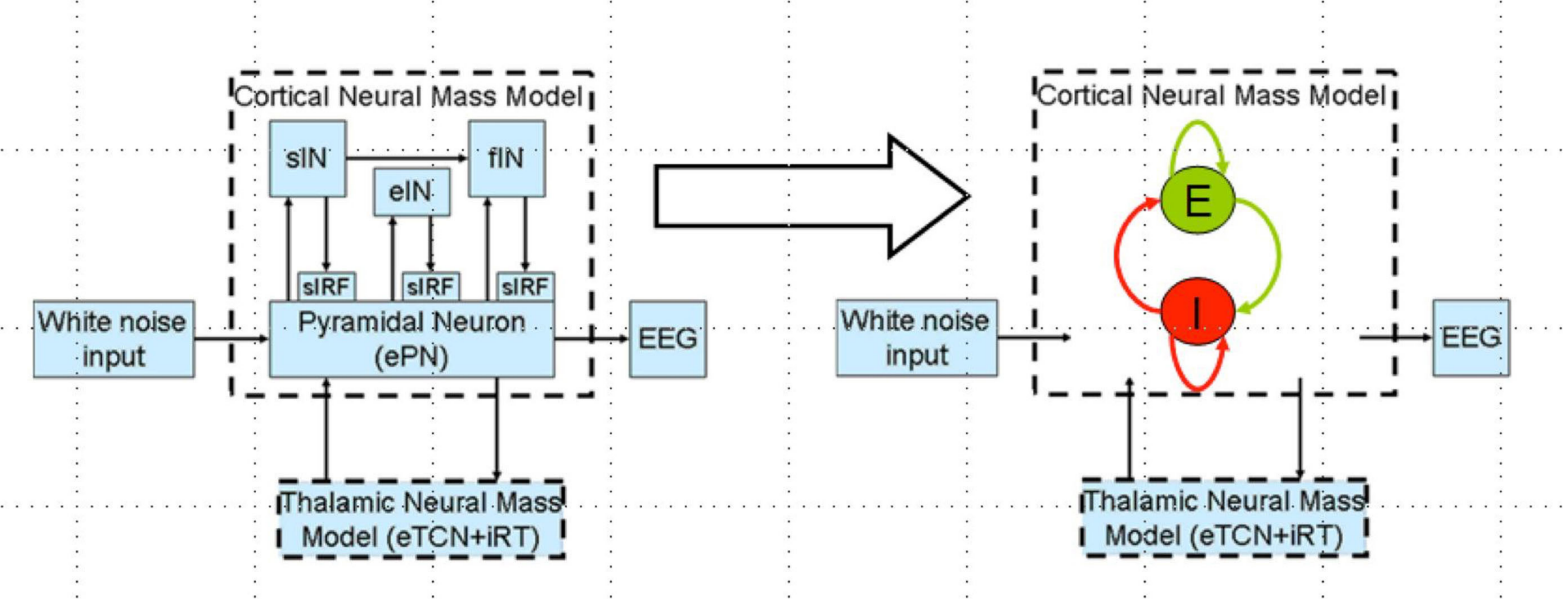
**Thalamocortical neural mass model (left panel) and a reduced network model of the cortical neural mass containing two populations (excitatory, E and inhibitory, I) of adaptive neural masses receiving recurrent external inputs from thalamic neural mass (right panel)**.

Polarity-specific cortical excitability alterations have been shown with transcranial direct current stimulation (tDCS) (3, 37) where it is postulated that the orientation of the electric field may be more relevant for neuronal stimulation while the electric field strength may be more relevant for astrocytic stimulation within the neurovascular unit (NVU) (16). After the forward model of the electric field is obtained (see Figure 2A), the sensitivity of the neuronal population (or, neural mass) to the electric field is determined by its morphology (38). Such tDCS-induced neuronal membrane polarization in a polarity-dependent manner can lead to synaptically driven after-effects after a sufficient long stimulation duration (39). The transmembrane currents, primarily responsible for EEG, contribute to intrinsic resonance and fluctuations of the membrane potential where inter-neurons and thalamocortical inputs can play a significant role in shaping the power spectrum (21, 36, 40). In fact, these spatiotemporal field fluctuations in the brain may “feedback” (and even amplify) the cellular discharge properties thereby shaping the power spectrum (40). Here, NMM can provide insights into the neuromodulatory mechanisms underlying alterations of cortical activity induced *via* tDCS (40). In our prior work, we explored the origin of tDCS-induced alterations in the electroencephalogram (EEG) power spectrum using a thalamocortical NMM (21). The left panel of the Figure 3 shows a single cortical source NMM coupled to a thalamic NMM. The cortical NMM comprises of ePN, excitatory interneurons (eIN), slow inhibitory interneurons (siIN), and fast inhibitory interneurons (fiIN) based on prior work (41). Also, the thalamic NMM comprised an excitatory thalamocortical (eTCN) and an inhibitory reticular-thalamic (iRT) based on prior work (42). The population of ePN (output) cells receives inputs from inhibitory and excitatory populations of interneurons *via* intrinsic connections (intrinsic connections are confined to the cortical sheet). An extrinsic thalamo–cortico–thalamic loop consists of eTCN and iRT in the thalamic NMM (43). We found that anodal tDCS non-specifically enhanced the activity of the ePN at a population level where μ-rhythm desynchronization was generated in EEG. Also, the modifications to the model parameters (e.g., average gain of synapses, their time constants) (41) of the lumped thalamo–cortico–thalamic network model was used to successfully simulate the subject-specific EEG power spectral density changes during/following tDCS (21). Here, the excitation versus inhibition effects of acute tDCS on the population kinetics can produce a whole spectrum of EEG signals within the oscillatory regime of the NMM (44) so that the cortical NMM can be sub-divided into two populations – excitatory, E, and inhibitory, I, – of adaptive neural masses (right panel of Figure 3). At a macroscopic level, Radman et al. quantified the cell-specific polarization by weak direct current fields using a “coupling constant,” which is a single number linearly relating the membrane polarization at any given compartment, including the soma, with the stimulation intensity. Therefore, different sub-populations of neurons will be affected differently by a given stimulation protocol, which may have distinct affects on E–I neuronal populations/neuronal compartments. For example, the Layer V pyramidal neurons exhibit the highest measured somatic sensitivities to subthreshold fields (45) such that somatic depolarization of Layer V pyramidal neurons by anodal tDCS may result in corresponding alterations of spontaneous firing rate (38) thereby causing a rapid increase in extracellular ionic concentrations that can activate the glial network (46). The glial network plays an important role in regulating neural activity by spatial buffering with a time course of seconds (47). Due to this relatively long time course, some of these diffusing extracellular ions can act as mediators of vasodilation (48) as well as neurotransmitters, affecting other neuronal compartments, including GABAergic and glutamatergic synapses. Moreover, the large glial–vascular bath within the NVU that can buffer extracellular ion concentrations will result in an inhibitory mechanism (49) for the cortical NMM (26). Also, it has been postulated recently that tDCS can directly affect the astrocytic network within the NVU (16).

### Neurovascular Unit Model Coupling Hemodynamic Response Captured with fNIRS and Neuronal Response Captured with EEG during tDCS

**Figure 4:**
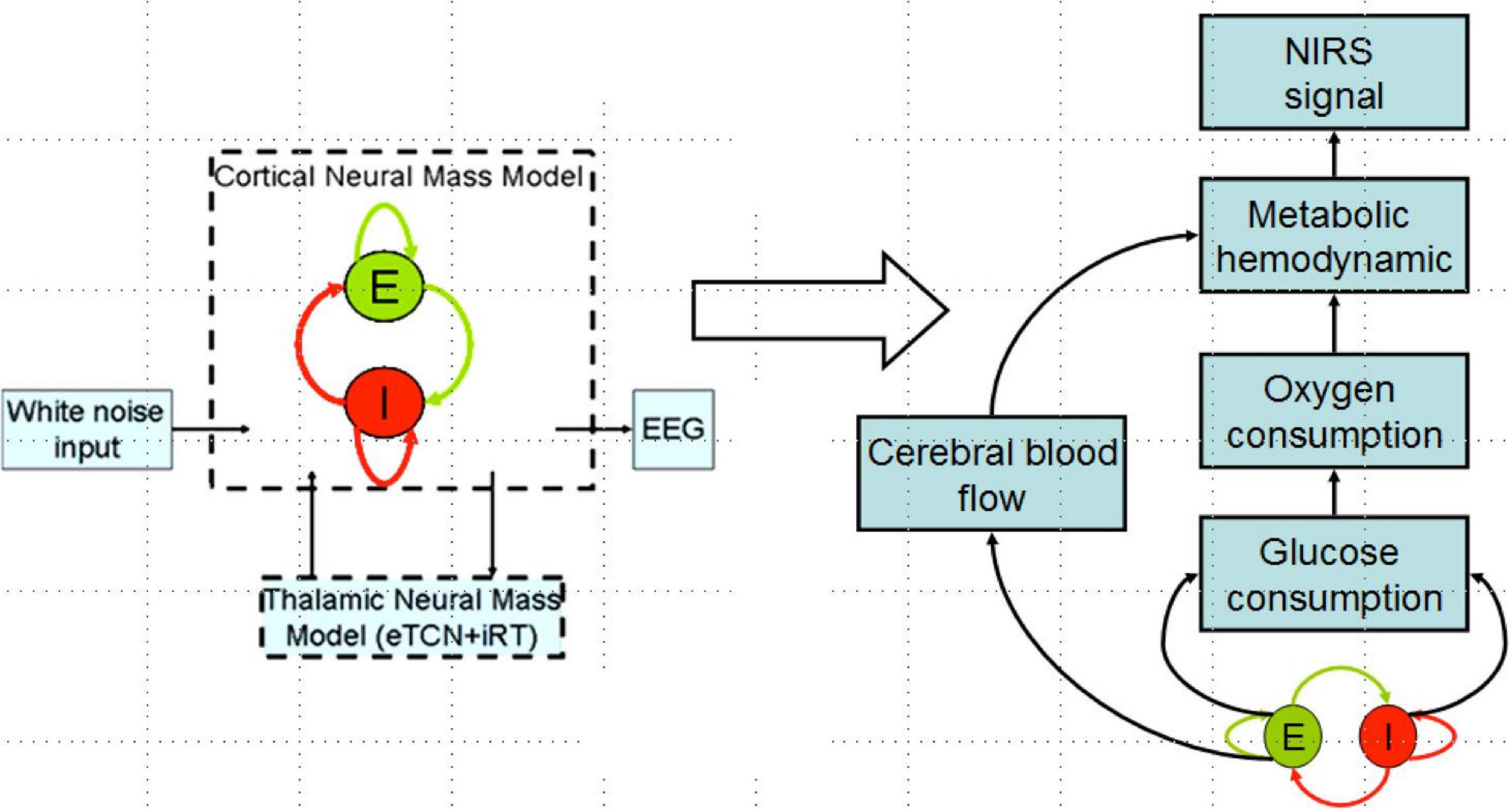
**Coupling hemodynamic response captured with NIRS with neuronal response captured with EEG for the cortical neural mass jointly imaged with simultaneous NIRS and EEG**.

Zheng et al. (50) have found a significant correlation between the strength of tDCS current and the increase in regional cerebral blood flow (CBF). This regional cerebral vascular reactivity (CVR), defined as the change in CBF to tDCS current (32), was investigated during anodal tDCS-induced local brain activation by adapting an arteriolar compliance model of the CBF response to a neural stimulus (51). Here, Dutta et al. (32) used continuous-wave NIRS to capture hemodynamic response which can be combined with diffusion correlation spectroscopy (52) to non-invasively measure cerebral blood flow as well as blood oxygenation (53). If the hemodynamic response to tDCS is captured with NIRS simultaneously with neuronal response using EEG, then the coupling relation via metabolic hemodynamics (54) can provide an estimate of the state of the neurovascular unit, as illustrated in Figure 4 and explained below. It was found that the tDCS-induced change in the synaptic transmembrane current, *u*(*t*) (only excitatory effects were considered in Dutta et al. (32) but both excitatory and inhibitory metabolic effects will be relevant) (40) can be captured by a first-order Friston’s model (55) relating *u*(*t*) to a change in the concentration of multiple vasoactive agents (such as NO, potassium ions, adenosine) causing a change in a single vascular flow-inducing vasoactive signal, *s*, leading to CBF. Oxygen consumption is limited by the diffusion of oxygen from the vasculature in case of diffusion-limited oxygen delivery (56), and thus oxygen consumption is tightly coupled to the induced blood flow (57) and the surface area of the vasculature. Here, the NMM (21) discussed in “Section Neural Mass Model for Capturing Neuronal Response to tDCS using EEG Related Anodal tDCS Intensity” (current density), σ(*t*), to the tDCS-induced changes in synaptic transmembrane current, *u*(*t*) (40), which was coupled with this phenomological hemodynamic model representing changes in the CBF. Now, neuroenergetics of the neural activity (both excitatory and inhibitory metabolic effects) can relate the hemodynamic (Hbt) response to anodal tDCS, σ(*t*), where deoxy- (Hb) was a byproduct of the consumption of oxygen delivered by oxy- (HbO_2_) hemoglobin that can be estimated using the cerebral metabolic rate of oxygen, CMRO_2_ (32). However, fNIRS is an optical imaging technique where a transfer function representing the sensitivity matrix of the optics equation can relate the optical density changes in fNIRS due to the changes in chromophores, HbO_2_ and Hb. Recently, Tak et al. (58) presented such a generative model for fNIRS data based on the interactions among hidden neuronal states that could be elucidated from EEG (59) with fNIRS–EEG joint imaging. fNIRS–EEG joint imaging is ideally suited with its spatiotemporal resolution to capture the cortical state of the NVU where tDCS has primarily cortical direct effects for its electric field penetration with spatiotemporal range amenable to fNIRS–EEG joint imaging (16).

### Interactions between Neuronal and Hemodynamic Responses to tDCS

**Figure 5:**
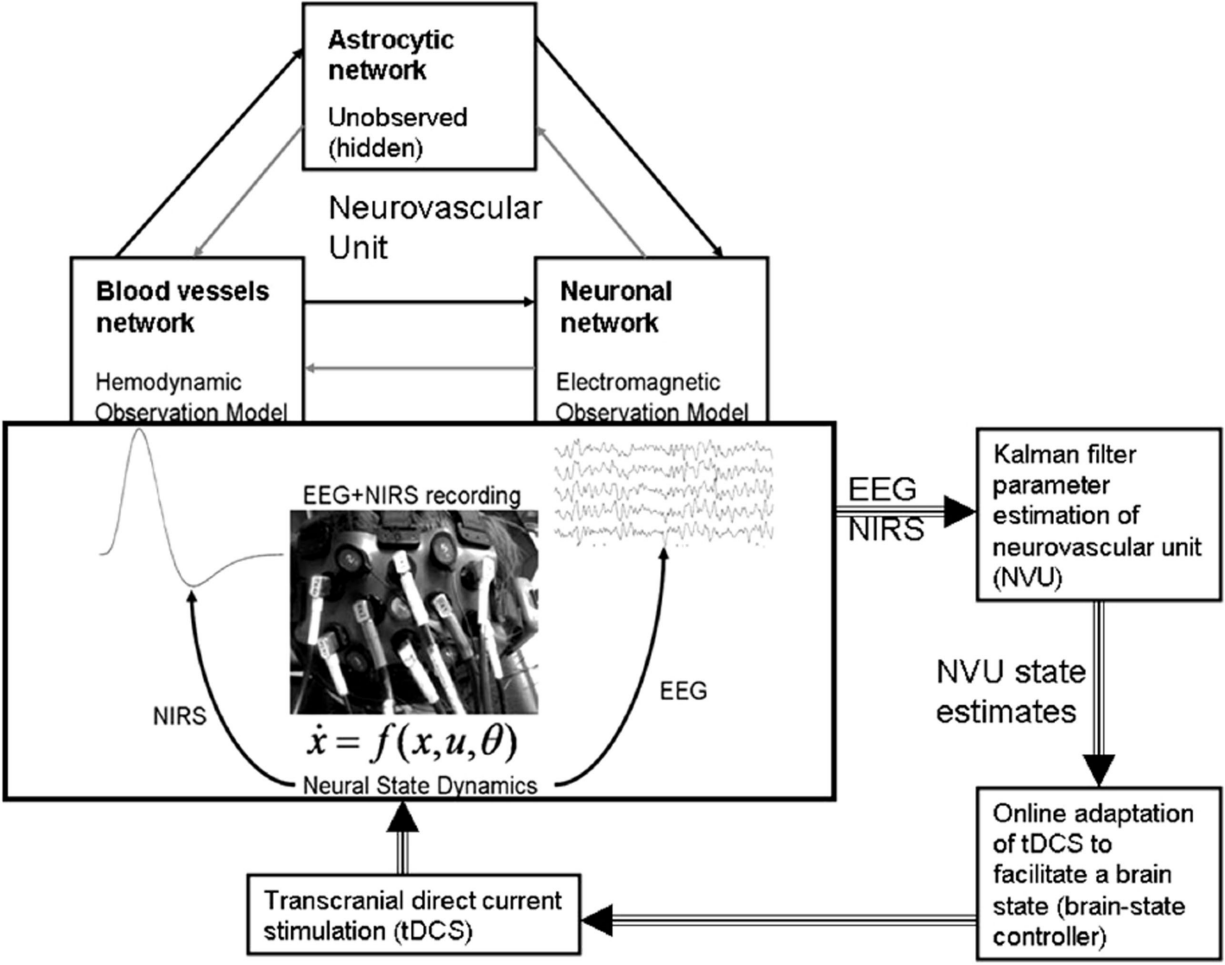
**Neurovascular unit (NVU) that consists of the endothelium, glia, neurons, pericytes, and the basal lamina**. Computational model for the interactions between the hemodynamic and electrophysiological responses, captured with NIRS–EEG joint imaging, may help in online modulation of tDCS. Figure adapted from Dutta et al. (19, 26).

The homeostasis of the brain microenvironment is maintained by the neurons, astrocytes, and vessels operating in tandem as semi-independent networks within the NVU (60) consisting of the endothelium, glia, neurons, pericytes, and the basal lamina (see Figure 5). It has been postulated that the neurons as a collective phenomena alter their intrinsic or synaptic properties to maintain a target level of electrical activity (61), which is called homeostatic regulation of neuronal excitability. In homeostatic regulation of neuronal excitability, previous amount of network activity determines the ease with which a synaptic connection is facilitated or suppressed (62–65). Here, Fricke and coworkers (62) hypothesized a role for L-type voltage-gated Ca^2+^ channels (L-VGCC) in short-term homeostatic plasticity, since tDCS has been shown to induce a long-lasting disturbance of Ca^2+^ homeostasis (66) and induce calcium-dependent plasticity (67). In principal accordance, Fricke and coworkers (62) proposed two ideas based on prior animal experiments to explain the time course of the induction of homeostatic plasticity generated by repeated tDCS of the human motor cortex [see Ref. (68, 69)] that the direction [long-term potentiation (LTP)/long-term depression (LTD)] of synaptic plasticity depends on the magnitude and dynamics of different postsynaptic levels of Ca^2+^ induced by the presynaptic input, with high levels favoring LTP and lower levels LTD; and (2) that the history of activation of a neuron can affect the function of L-VGCC channels such that high preceding levels of activity would reduce their activity, whereas low levels would increase it. LTP/LTD can be elicited by activating *N*-methyl-d-aspartate (NMDA)-type glutamate receptors, typically by the coincident activity of pre- and postsynaptic neurons (70), which function as calcium channels. Moreover, neuronal activity can trigger Ca^2+^ signals (66) in apposed glial cells, and glial Ca^2+^ waves can affect neurons (71). The glial network may have an important role (i.e., spatial buffering) in regulating neural activity by distributing ions (47). An influence of long-lasting disturbance of Ca^2+^ homeostasis *via* tDCS on the myogenic and the metabolic control of cerebral circulation cannot be excluded. Here, recent computational models (72) presented bidirectional interactions within the NVU (26) where a (delayed) “reverse” influence in the NVU from the vessel back to neuron *via* lactate is possible with lactate as a signaling molecule (73). Indeed, recent work showed that lactate can modulate the activity of primary cortical neurons through a receptor-mediated pathway (74), and vasomotion rhythms can influence neural firing patterns (75) presenting interactions between neuronal and hemodynamic responses.

## E–I Balance Hypothesis for Brain-State Dependent Electrotherapy in Stroke Rehabilitation

During stroke rehabilitation, we postulate that peripheral NMES combined with tDCS would not only suppress maladaptive plastic changes but also facilitate beneficial neuroplasticity. Indeed, it was found that EMG-driven NMES affected greater brain cortical perfusion than voluntary muscle contraction or NMES alone (6) where the beneficial brain activation can be further facilitated with tDCS (7) toward beneficial neuroplasticity. Therefore, it is postulated that a tDCS in conjunction with peripheral electrical stimulation will modulate the activity in the perturbed network, to restore an adaptive equilibrium for optimal behavioral outcome and suppress maladaptive plastic changes for functional advantage *via* their synergistic effect on task–relevant neuronal activation patterns. Following initial human studies by Nitsche and Paulus (37), numerous subsequent tDCS studies have been performed using their conventional montage and stimulation parameters. However, recent studies have suggested that stimulation parameters may affect the focality and specificity of tDCS in inducing neuroplastic alterations (76). Moreover, in poststroke subjects, heterogeneously damaged cortical regions presents a challenge because of alterations of current flow, where individualized tDCS protocols based on neuroimaging are required (3). Therefore, computational techniques are required to develop patient-specific multi-electrode tDCS montages based on neuroimaging to optimize tDCS of the targeted brain locations (77).

### Computational Models to Understand Interplay between E–I Balance and Brain State Dependency during Electrotherapy

**Figure 6:**
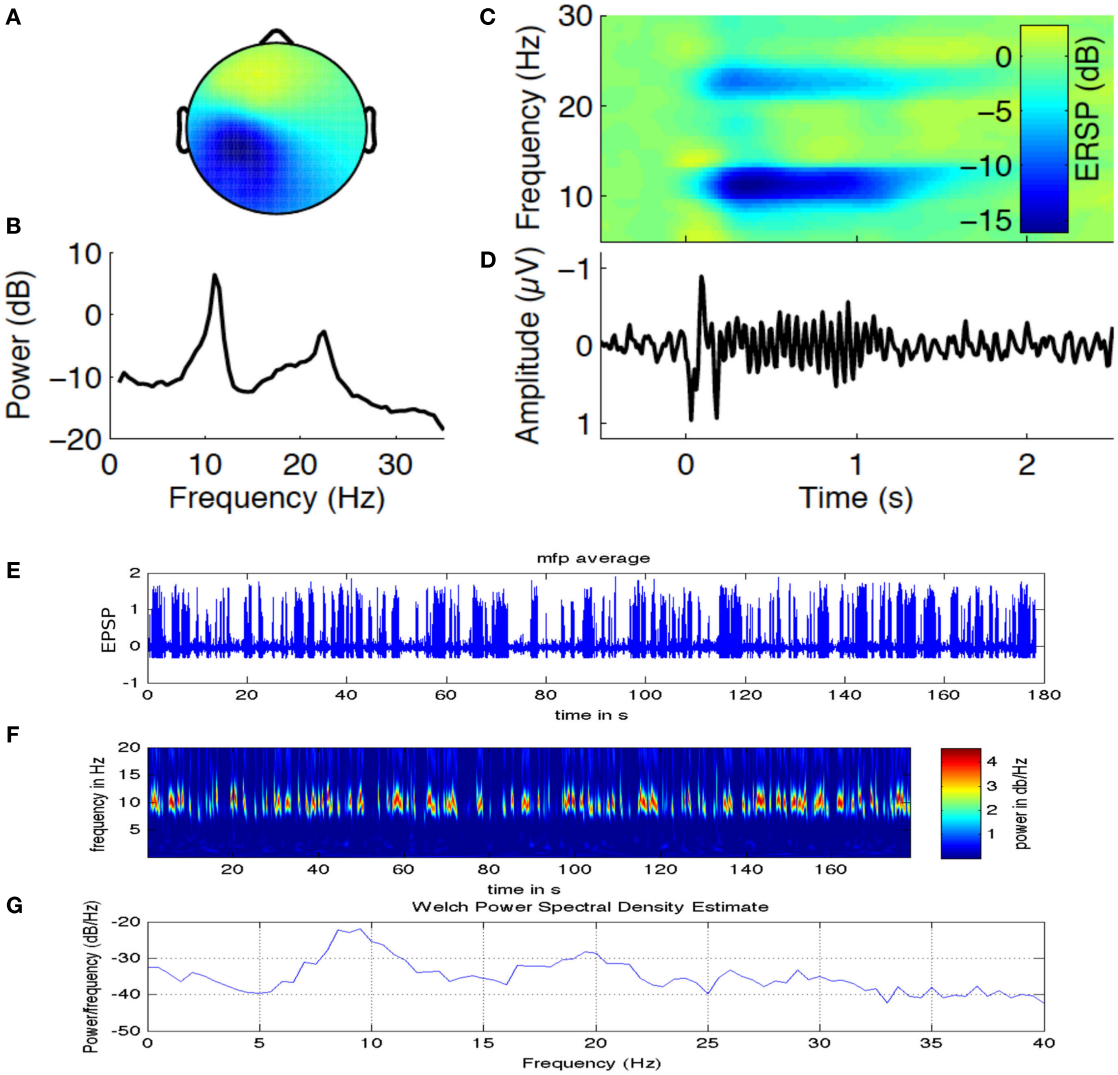
**Alpha band oscillations and state dependent reorganization in the cortex**. Figure adapted and modified from Sigala et al. (35). **(A)** Alpha and Beta band power in channels distributed over left somatosensory, motor cortex (contralateral side to the tactile stimulation side). **(B)** Two prominent power spectral peaks one at 10–12 Hz (central μ Alpha) and the other at 15–20 Hz (Beta band). **(C)** The evoked somatosensory response potential (ERSP) to repetitive stimulation where maximum powers in decibel are located in the two identified frequency bands. **(D)** Temporal dynamics of amplitude exhibit ERD followed by stimulation. **(E)** Simulated mean field EEG activity is shown for the somatosensory, motor cortex based on channel location map. **(F)** A representative example of Alpha band specific cortical activation. **(G)** Power spectral density estimate using Welch method to compare power spectral distribution from model against empirical power spectral distribution displayed in **(B)**.

In a recent study by Sigala et al. (35), authors have explored the possibility of using repetitive tactile stimulation protocol to explore the reorganization of resting state connectivity in the somatosensory cortex that is highly modulated by Alpha band power. Figure 6 is adapted from this study where we look at the EEG Alpha band power (indicating current brain state) and ERD generated over the channels located in the contralateral side of the stimulation on the left primary somatosensory cortex. Figure 6A captures the Alpha and Beta band power activation for a time frequency analysis in channels located in CPz and Cz distributed over left somatosensory, motor cortex and also partly association area. Figure 6B captures two peaks in the power spectra one at 10–12 Hz (central Alpha) and the other at 15–20 Hz (Beta frequency band) related to tactile, motor cortex stimulation. Figure 6C shows the evoked somatosensory response potential (ERSP) to stimulation where maximum powers in decibel are located in the two identified frequency bands. Figure 6D shows amplitude of the evoked response potential, which captures the ERD followed by stimulation. In Figure 6E, we have simulated a thalamo-cortical NMM similar to the one presented here in this article to generate mean field EEG activity in the somatosensory, motor cortex. Shown here in Figure 6F is a representative example of wavelet analysis on the generated EEG time series to reveal Alpha band specific cortical activation. Finally, in Figure 6G, we show power spectral density estimate using Welch method to compare power spectral distribution from model against empirical power spectral distribution displayed in Figure 6B. Power spectral density from computational model reveals ERSP (in dB) exhibits maximum in the Alpha and Beta frequency band as observed in the experimental results.

**Figure 7:**
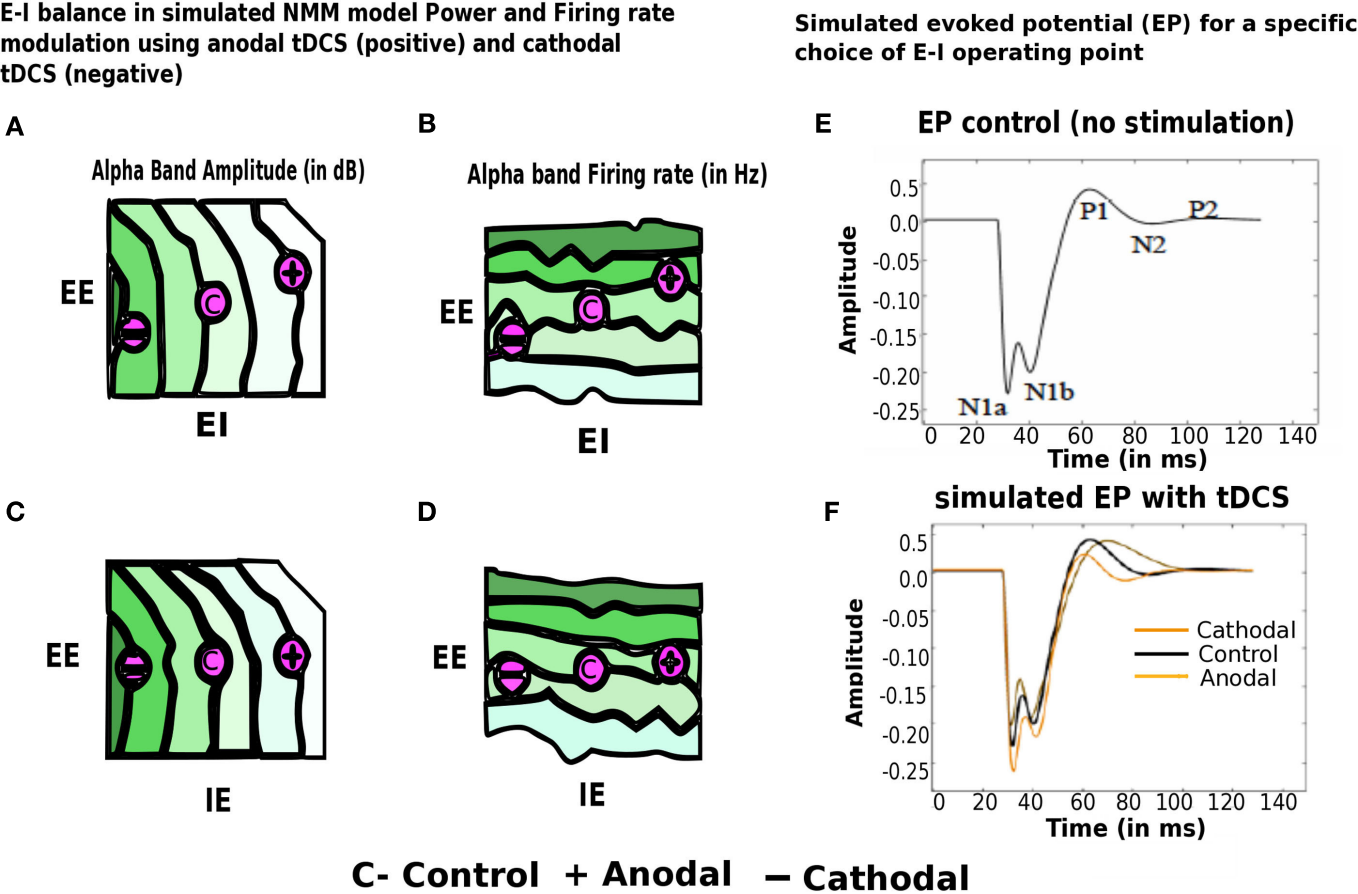
**Modulation of alpha band (brain state) amplitude, firing rate, EP with anodal and cathodal tDCS and EE, EI, IE synaptic parameters**. In **(A–D)** EI or IE synapses strongly modulated alpha band amplitude, power (10– 12 Hz) (highest values of amplitude, firing rate is color coded in dark green; lowest values of the same are color coded in white). Population firing rate is more sensitive to changes in EE connections. Points in this parameter space that are consistent with the present experimental observation are indicated with an “C” for control (no stimulation) and “+” for anodal (positive) and “−” cathodal (negative) electric field stimulation. In **(E–F)**, we look at the evoked potential (EP) under simulated control conditions (in black no stimulation) and also simulated with cathodal (in dark yellow), anodal electric field (in light yellow), stimulations, respectively [figure adapted and modified from Molaee-Ardekani et al. (40)].

Next, we present preliminary results based on simulation of computational NMM. Our preliminary investigations suggest that modulation of feedback inhibitory synaptic strength plays critical role in the model. Recent evidence for plasticity in inhibitory connections (78) motivates underlying biophysical parameter space explorations. Role of inhibition is further demonstrated in a computational cortical model proposed recently by Reato et al. (14) and Vattikonda et al. (27), a thalamo-cortical mean field model by Roy et al. (36). Our computational results show that the modulation of strength of specific synapses changing (EI) or (IE) synapses strongly modulated Alpha band amplitude, power (10–12 Hz) (Figure 7). Average firing rate of excitatory units are more sensitive to changes in EE connections (Figure 7). Steady state solutions in the parameter space that concurs with the present experimental observation are indicated with an “C” for control (no stimulation) and “+”, “−” for anodal (positive) and cathodal (negative) electric field stimulation. Interestingly, along these diagonals, variables such as amplitude, power and firing-rate change while oscillatory frequency remains the same. Therefore, the computational results suggest that the observed sustained changes may be explained partly by the alteration of excitation matched by a corresponding change in feedback inhibition (a kind of homeostatic control mechanism). In Figure 7, we look at the evoked potential (EP) under simulated control conditions and also simulated with cathodal, anodal electric field stimulations, respectively. This preliminary result suggests naively the operating point in the EE–EI or EE–IE parameter space that may bring the EP response closer to control whenever excess runway excitation is resulted from positive or more depression resulted from negative stimulation.

Transcranial direct current stimulation differs qualitatively from other brain stimulation techniques such as transcranial electrical stimulation (TES) and transcranial magnetic stimulation (TMS). In case of tDCS, static fields in the stimulation range do not yield the rapid depolarization required to produce action potentials in neuronal membranes. Hence, tDCS might be considered a neuromodulatory intervention. In this stimulation, exposed tissue is polarized, and tDCS modifies spontaneous neuronal excitability and activity by a tonic de- or hyperpolarization of resting membrane potential (42, 79). The efficacy of tDCS to induce acute modifications of membrane polarity depends on current density, which determines the induced electrical field strength (79) and is the quotient of current strength and electrode size. Also, for humans, it was shown that larger current densities result in stronger effects of tDCS. Due to very weak currents and the non-invasiveness of tDCS, this technique is suitable for modulation of the cerebral cortex, the most outer part of the brain, which lies closest to the surface electrodes attached to the patient’s head. This weak direct current applied non-invasively is strong enough to elicit significant effects on cortical activity. These effects cause the oxygen saturation levels to increase at the stimulated area in the healthy case. It has been shown that anodal tDCS enhanced activity and excitability of the ePN at a population level in a non-specific manner, where μ-rhythm de-synchronization found to be generated (43). Studies suggests that cathodal tDCS decreases the firing rate of neurons thereby down-regulating the activity and excitability of the ePN (43). Cathodal tDCS induces a decrease in regional CBF (rCBF) in cortical and subcortical areas (44). tDCS involves the use of at least two surface electrodes (one anode and one cathode) to deliver a stimulating current to the patient. Electrical current flows in the direction from anode to cathode for anodal tDCS. It induces polarity-specific changes of cortical blood perfusion (45). In fact, a significant correlation between tDCS current strength and increase in rCBF has been found (46) that can be captured using NIRS [Diffusion Correlation Spectroscopy (52)]. It has been postulated that CBF-alterations are causally related to tDCS-induced alterations in cortical excitability *via* neuro-vascular coupling. Anodal tDCS can increase rCBF during stimulation. Thus combining NIRS with tDCS can be an easy and economical setup for use in clinical population at risk for ischemic stroke (80).

### Brain-State Dependent Adaptive tDCS System

**Figure 8:**
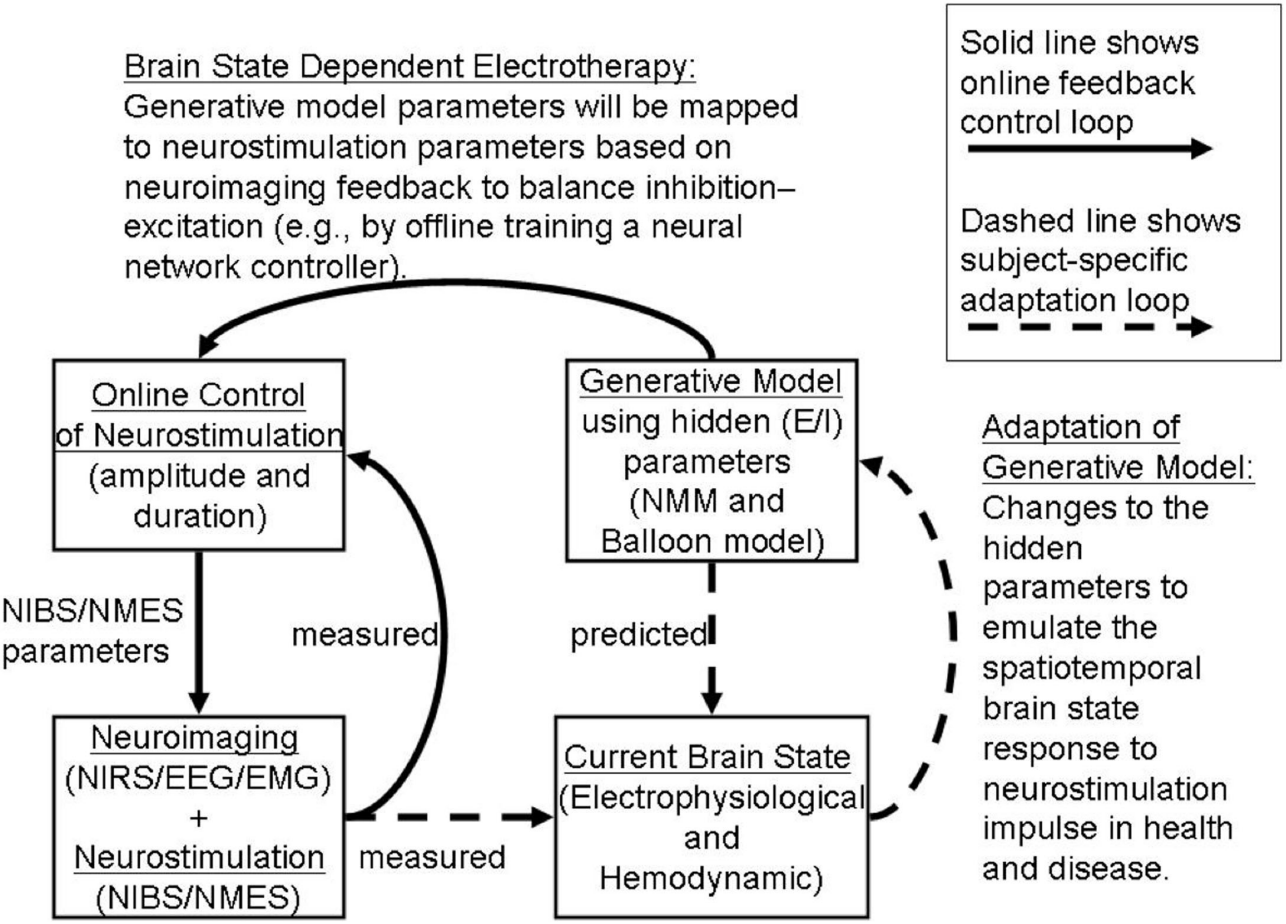
**Adaptive Brain State Dependent electrotherapy (BSDE)**. The block diagram depicts combined recording of brain signals from fNIRS and EEG. Brain signals recorded during online tDCS convey relevant EEG and Hemodynamic parameters. This parameter set provides the current brain state. An independent block diagram is presented in the form of a neural mass model (NMM). Output neuronal signal convolved with a Balloon model to generate Hemodynamic response and EEG forward model is used as a generator of EEG activity from specific channel location where stimulation is applied. Generative model parameters are adjusted and calibrated based on the comparison between simulated and measured current brain states. Generative model estimates the predicted E/I state as shown in the block diagram. Predicted E/I states are compared with a Multilayer Neural Network (NN) model that is pre-trained offline with control signals taken from healthy and patient populations. tDCS stimulation parameters are derived in the online control module by arriving at a match between the predicted E/I and measured E/I values. This stimulation parameter values are then used for application of tDCS.

We propose an online tDCS adaptation system, called Brain State Dependent electrotherapy (BSDE) system, whose central aim is achieving E–I balance for therapeutic intervention (see Figure 8). The hemodynamic and electromagnetic variables corresponding to tDCS stimulation are estimated from the combined fNIRS and EEG system (as shown in Figure 5) using the framework developed by Dutta et al. (19). The Neural Mass Model system works as a generative model that takes the variables estimated from fNIRS and EEG such as ratio of oxygenated-to-deoxygenated hemoglobin, band-specific power, event-related de-synchronization (ERD), etc., in order to compute the E–I balance state corresponding to the current stimulation epoch. The estimated state from the NMM system is used along with the current stimulation parameters (such as amplitude of stimulation current and the duration of stimulation) as inputs to a trained model in order to predict the required parameters for anodal and cathodal tDCS. These predictions are used in conjunction with the control chart shown in Figure 7 to arrive at optimal stimulation protocol to achieve target E–I balance. Trained model that maps current E–I state and stimulation parameters to the target values is constructed off-line using data from patient and healthy controls. The proposed closed-loop system is aimed at online adaptation of the tDCS stimulation protocol for efficient and effective therapy.

## Discussion

Because the state of brain networks is more likely to be altered depending on the impact of damage, perturbation to neural state; the stimulation strategies that work fairly accurately in healthy control may need to be adapted for clinical applications. In this perspective article, we have proposed brain state dependent NIRS-EEG neuroimaging of those brain networks during NIBS and an understanding of operating point based on computational models. To address this systematically interactions between a particular brain state and E–I balance in a group of neurons located in a target brain area during focal perturbation is absolutely necessary to identify the operating point of the parameter space. Recent *in vivo* and *in vitro* studies have demonstrated that the electric fields whose amplitude is comparable to the one expected in NIBS, can modulate firing rate (81), Spike timing (82), and the magnitude of the synaptic responses (45). Reato and coworkers (14) have shown that acute effects of weak electrical stimulation can be amplified during endogenous oscillatory activity. Weak constant current electrical stimulation applied for a longer period of time can induce lasting effects, measureable potentially as altered Gamma frequency band power and multi-unit activity (MUA). Importantly, this poststimulation effect was consistent with the acute effect, reminiscent of Hebbian like activation or neuroplastic effects. In this article, we also propose an online, adaptive, closed-loop control framework for NIBS whose central objective is to restore E–I balance that has been perturbed following neural impairment. Online balancing of E–I is achieved by matching the current brain state estimated from fNIRS and EEG with that predicted by a generative computational model comprising neural masses. Subsequently the predicted E–I state is compared with a target operating point in order to adjust the stimulation protocol. Our proposed framework for a BSDE combined with generative large-scale biologically realistic computational models is presented here in the form of an E–I balance hypothesis (see Figure 8). We expect that numerous testable predictions would come out from our approach that not only will benchmark our model as a tool for clinical research but also would provide systematic insight about the limitation of certain existing protocols with regards to NIBS. Independent of this study, a computational modeling work constrained by combined EEG–fNIRS imaging (16) is currently underway to test the limitation and success of this framework as an adjuvant therapeutic approach to improve efficacy of rehabilitation.

## Author Contributions

DR and AD designed the study. SD, SC, DR, AD, and RB carried out research. SD, RB and DR contributed in the computational work. AD and SC carried out hardware design, experimental design and protocol. SD, RB, SC, AD, and DR all contributed in preparing and writing manuscript.

## Conflict of Interest Statement

The authors declare that the research was conducted in the absence of any commercial or financial relationships that could be construed as a potential conflict of interest.
